# A Comprehensive Review of Continuous Glucose Monitoring Accuracy during Exercise Periods

**DOI:** 10.3390/s21020479

**Published:** 2021-01-12

**Authors:** Elena Muñoz Fabra, José-Luis Díez, Jorge Bondia, Alejandro José Laguna Sanz

**Affiliations:** 1 Instituto Universitario de Automática e Informática Industrial, Universitat Politècnica de València, Camino de Vera s/n, 46022 València, Spain; emunozfabra@gmail.com (E.M.F.); jldiez@isa.upv.es (J.-L.D.); 2 Centro de Investigación Biomédica en Red de Diabetes y Enfermedades Metabólicas Asociadas (CIBERDEM), Instituto de Salud Carlos III, 28029 Madrid, Spain; allasan@upvnet.upv.es

**Keywords:** continuous glucose monitoring, type 1 diabetes, physical activity, accuracy, exercise, mean absolute relative difference

## Abstract

Continuous Glucose Monitoring (CGM) has been a springboard of new diabetes management technologies such as integrated sensor-pump systems, the artificial pancreas, and more recently, smart pens. It also allows patients to make better informed decisions compared to a few measurements per day from a glucometer. However, CGM accuracy is reportedly affected during exercise periods, which can impact the effectiveness of CGM-based treatments. In this review, several studies that used CGM during exercise periods are scrutinized. An extensive literature review of clinical trials including exercise and CGM in type 1 diabetes was conducted. The gathered data were critically analysed, especially the Mean Absolute Relative Difference (MARD), as the main metric of glucose accuracy. Most papers did not provide accuracy metrics that differentiated between exercise and rest (non-exercise) periods, which hindered comparative data analysis. Nevertheless, the statistic results confirmed that CGM during exercise periods is less accurate.

## 1. Introduction

Type 1 Diabetes (T1D) is a health condition in which insulin secretion by the pancreas is impaired or is completely missing, causing high levels of blood glucose in the affected patients. According to the International Diabetes Federation (IDF) [1], seven-point-seven billion people in the world population have diabetes. Of this figure, one-million, one-hundred-ten-thousand, one-hundred children and adolescents are known to suffer from T1D. In general, it can be estimated that around 5–10% of all diabetes cases correspond to T1D. This clearly has a great economic impact, such that by 2019, the IDF estimated that total diabetes-related health expenditure reached USD 760 billion.

Glucose homoeostasis is maintained naturally for individuals without diabetes, but people with T1D must control blood glucose concentration daily by means of exogenous insulin delivery. In recent years, several medical devices have been introduced to facilitate the management of insulin treatments for T1D. Continuous Glucose Monitoring (CGM) systems are some of the most popular, which provide continuous information about glucose levels based on a subcutaneously inserted probe that estimates glucose concentrations in blood based on interstitial electro-chemical measurements.

CGM has allowed real-time detection of changes in glucose levels and its immediate control. Even though the use of CGM is widespread among people with T1D, several studies showed [2,3,4,5,6,7] an important lack of accuracy under rapid and unexpected glucose rate changes, as occurs while doing exercise, commonly increasing the risk of hypoglycaemia. As these studies suggest, an understanding of this loss of accuracy during exercise might lead to compensation schemes mitigating its impact [8].

In this report, original studies that use CGM in T1D patients during different types of exercises are collected and compared. Specifically, the Mean Absolute Relative Difference (MARD) is analysed as a metric of the overall accuracy of CGM, and the MARD difference between the rest and exercise periods of the patients are calculated (ΔMARD), which provide a differential measurement of performance for exercise with respect to the “rest” periods. Even though there have been publications that review and compare studies that use CGM during exercise [9,10], these do not overlap with our paper, as they do not specifically analyse CGM accuracy according to the MARD value obtained, and they do not compare MARD values during exercise periods and the rest counterparts.

In this paper, we first describe the methods and exclusion criteria used for selecting the chosen articles (Section 2); next, the studies selected are screened (Section 3.1). Then, the results (Section 3) are discussed (Section 4). Finally, the conclusion reached, due to the given information, is presented (Section 5).

## 2. Materials and Methods

### 2.1. Eligibility Criteria

In this review, articles that reported original data on the use of CGM systems in T1D patients during a period of exercise were screened, selected and analysed. No distinction on the age of the patients was made.

The essential data extracted from each article were: publication year, cohort information (number of patients, age and sex), type(s) of exercise, total number of samples during exercise, glucose sensor and reference used for the computation of MARD. For those studies that provided an MARD value for the exercise periods, the total number of samples (obtained as the accumulated number of samples during exercise for all the trial participants) was extracted, and for those that also provided the MARD during rest periods, these data were also registered. Exercise intensity (% VO2 max) and duration (min) were also extracted for the analysis.

Discrepancies about the papers were resolved through consensus. Those articles that did not or not explicitly report the outcomes of interest were excluded.

Every study selected was classified, after critical scrutiny of the exercise description therein, into one of the next types:

- Aerobic: Patients usually exercised cycling (cycloergometer) or walking on a treadmill completing bouts during an amount of time and certain intensity of a fraction of the patients’ maximum capacity. Rest time was also registered.
- Resistance: Patients exercised doing bouts of weight lifting exercises, ensuring that major muscles groups were targeted (leg press, bench press, leg curl, lat pull-down, abdominal crunches, shoulder press, seated row, etc.). Exercise was performed at a certain intensity and establishing the time between one bout and the next one.
- High Intensity Interval Exercise (HIIE): Patients exercised doing periods of maximum intensity exercise, usually involving very fast repetitions, alternated with short periods of resting or low-to-moderate intensity exercise. This type of exercise usually ends with exhaustion, and protocols can be very diverse.
- Intermittent: Patients exercised in intervals in which the intensity of the activity varied between different levels. Each interval is repeated cyclically until a determinate resting time is reached. For example, a one hour light running exercise with periodic bouts of faster sprint reaching 80% VO2 max every 10 min would qualify as intermittent.

A classification based on the method used to compute reference glucose for the computation of the MARD was also carried out:

- Gold standard: measurements were made in the laboratory by a glucose analyser from blood samples (Yellow Springs Instrument (YSI) or an equivalent device).
- Self-Monitoring Blood Glucose (SMBG): a glucometer was used involving fingerstick measurements.

MARD measurements for both the exercise and rest periods were the primary outcome extracted from the screened work to ensure that a proper statistical analysis of the data could be made and the accuracy of the CGM devices could be determined. When the MARD was not reported during the rest period, it was obtained (for the same sensor) from other articles, the main purpose of which was to study a determinate sensor accuracy without inclusion of exercise sessions [11,12].

### 2.2. Search Methods

An exhaustive electronic search was performed by using the National Center for Biotechnology Information (NCBI) library, called the National Library of Medicine (NLM) (including PubMed and PMC) [13]. The search strategy consisted of articles that included the keywords: CGM, continuous glucose monitoring, real time glucose monitoring, type 1 diabetes, exercise, physical activity, aerobic, resistance exercises, MARD. Once an article was selected and analysed, its references were examined, as long as they also included the same keywords. Furthermore, the recommendations provided by the web in the section “Similar Articles” were also revised. No language filter or date restrictions were applied.

### 2.3. Study Selection

Following the initial search, the title and abstract of the articles were analysed, and those that matched the inclusion criteria (Section 2.1) were further explored by thoroughly reviewing the nature of the studies described. Then, the content of the full document and the data confirmed its selection. Accuracy metrics were then extracted and stored for each of the selected studies.

### 2.4. Statistical Methods

Mean and SD values for the MARD during exercise and rest periods were extracted from the selected publications. Since clinical and statistical heterogeneity was expected among studies, a random effects model meta-analysis was conducted using inverse variance weighting for pooling [14,15,16]. The Hartung–Knapp method (also proposed by Sidik and Jonkman) was used for this purpose, since superior accuracy has been reported, especially when the number of studies is small [17,18]. The analysis was implemented in RStudio (RStudio Boston, MA, USA) using the function metacont, which was configured to use the standardised mean difference as the summary measure (Hedges’ method [19]), the Sidik–Jonkman method for the estimation of between-study variance, and the computation of prediction intervals with the method proposed by Higgins et al. [20]. Furthermore, subgroup analysis was performed using the RStudio metamean function, which computes the overall mean from studies reporting a single mean using the inverse variance method for pooling. The function was configured to use the method described in Luo et al. [21] to estimate missing sample mean values from the reported sample size, median, range, and/or interquartile range. All statistical hypothesis were tested at a 95% confidence level (α=0.05).

## 3. Results

### 3.1. Studies Selected

The search produced several studies relevant to the aforementioned goals: a total of 54 sources. The data were extracted and are summarized in Table 1, in order to provide the reader with a snapshot view of the CGM-exercise literature.

The oldest study (2011) that reported CGM accuracy is Adolfsson et al. [4], in which the reported accuracy metric, the MARD, ranged between 19% and 27%. A total of 24 adolescents were tested in three different sports (soccer, golf and floorball) using SMBG as the reference. The sensor used was the CGMS with MiniMed Solutions software Version 3.0.

A few years later, in 2016, Moser et al. [33] presented a study that also used the SMBG method for glucose reference. Eight patients were tested using a cycle ergometer executing continuous and High Intensity Interval Exercise (HIIE) protocols, using the Guardian Real-Time. The MARD exercise remained in the same relative range (18–20%). In the same year, Bally et al. [34] also presented the MARD exercise within a good range (13.3% for intermittent and 13.6% for aerobic). They used the Dexcom G4 Platinum with YSI 2300 STAT Plus Glucose Analyser to calculate sensor accuracy. Jayawardene et al. [39] involved 12 patients in a trial that explored the impact of aerobic exercise (cycling), reporting a 9.9% MARD; and HIIE exercise with 10.5%, using also a YSI Analyser, but with the Medtronic Minimed 670G, which integrates the Guardian Sensor 3 as the sensing unit. Although this review focuses on sensing accuracy, the name of the integrated sensor-pump systems will still be used throughout the manuscript to keep faithful to the device description in the source papers. However, a codification will be added to highlight the sensor incorporated. Thus, this device will be referred to as Medtronic Minimed 670G-S3.

One of the most recent studies selected in this review is Fokkert et al. [67]. Fourteen patients were monitored during six days of mountain biking activity, using the Guardian-Connect and the FreeStyle Libre sensor, reporting a higher MARD than other studies for the exercise period, with values of 29% and 22%, respectively. On the other hand, Guillot et al. [68], which is also a recent study, presented a lower MARD for the three types of exercise, using the Dexcom G6 and SMBG to calculate the CGM accuracy.

Moser et al. [63] (2020) explored the FreeStyle Libre sensor accuracy on a cohort of 14 patients. The reported MARD value during exercise was higher than those previously mentioned: 29.8%. The MARD during the rest period was significantly lower, at 8.6%. This is one of the few studies found that reported both MARD values, which enables the possibility of a more detailed analysis of the impact of the MARD on exercise, since CGM accuracy can be normalized with respect to its rest counterpart, i.e., a ΔMARD can be computed. That is also the case for the study by Taleb et al. [35], which is also interesting since it studied the accuracy of two different sensors: Dexcom G4 Platinum, the data of which will be referenced as Taleb Dexcom in this analysis, and Paradigm Veo (Enlite 2), which will be referenced as Taleb Enlite. The integrated system composed of the Paradigm Veo and the Enlite 2 sensor, from now on, will be referred to as Paradigm Veo-E2. Both CGM systems were used in the same conditions by 17 patients. Aberer et al. [37] also studied three different sensors: FreeStyle Libre, Dexcom G4 Platinum and Medtronic MiniMed 640G (integrating the Enlite 2 sensor), but they only provided accuracy values for the exercise periods. This device will be referred to as Medtronic Minimed 640G-E2. Moser et al. [49] also tested multiple devices, recruiting 10 participants for aerobic exercises, using the iPro2 and MiniMed 640G-E2 in the same conditions. Steineck et al. [50] compared two different placements of Dexcom G4 on either the abdomen or the arm.

Giani et al. [6] explored the behaviours of 17 patients during intermittent exercise using the FreeStyle Libre, but with the particularity of reporting the MARD for both SMBG and YSI Analyser. It must be noted that the data from this study yielded negative ΔMARD values, which seems to contradict the general behaviour of CGM during exercise. This particularity will be further discussed in Section 4. Only one other study presented this issue, the result of resistance exercise in Zaharieva et al. [40]. From this article, it is worth noting the small standard deviations reported, ranging between 0.06 and 0.12 mg/dL. In both cases, twelve patients were monitored using the Paradigm Veo-E2 system with iPro2.

In Li et al. [3], six patients were monitored using the Paradigm Veo-E2, and it was one of the few studies that implemented HIIE as the exercise protocol, using Dexcom G4 Platinum to monitor 17 patients. The reported value for exercise was 17.8%, and ΔMARD was 7.4%, quite similar to Biagi et al.’s [46] data during aerobic exercise. However, the data from the same study [46] during resistance exercise reported a much lower ΔMARD.

In most of the mentioned articles, the CGM devices were calibrated using SMBG. Those that used FreeStyle Libre were already factory-calibrated, for example Giani et al. [6] and Aberer et al. [37]. Some articles did not specify the calibration method used, like Moser et al. [55], Williams et al. [63], Fokkert et al. [67] and Guillot et al. [68].

For the sake of completion, accuracy data for sensors used in studies that did not provide the MARD for rest periods were searched in complementary articles. An example of this is Mastrototaro et al. [69], which tested the accuracy and efficacy of the Guardian Real-Time CGM in 72 subjects, obtaining retrospectively from an open-label, multicenter, six month study. Slover et al. [70] used a fourth-generation sensor, the Guardian Connect, tested in 145 patients, and Rodbard et al. [71] summarized some of the current CGM systems from Medtronic, including the integrated system MiniMed 640G with the Enlite sensor. Nakamura et al. [72] monitored 72 subjects who were enrolled at four US centres, wore the Dexcom G4 Platinum for up to seven days, and participated in a total of 36 h of monitoring, using YSI Analyser. Garg et al. [73] studied 30 adolescents and 94 adults using MiniMed 670G-S3 with a reference measurement by an i-STAT device. Gross et al. [74] monitored the glucose of 135 patients from eight clinical sites using the CGMS during patient home use for three days or more.

Complementary articles were matched only by the type of sensor used. Other factors that could affect sensor accuracy, such as age [75] or protocol characteristics, were unfeasible to be matched, and furthermore, it was desired to maintain a simple relationship between paired MARD values. However, to avoid confounding factors, only studies reporting both MARD during exercise and rest periods were included in the ΔMARD meta-analysis study. Matched articles are marked with an asterisk in Table 1. These data are organised in Table 2.

**Table 2:** Reported MARD data for resting periods for the sensors that the studies in Table 1 did not originally provide.

Paper Extracted	Sensor Used	MARD Rest (%)	Reference
Gross et al. [74]	CGMS	18	SMBG
Mastrototaro et al. [69]	Guardian Real-Time	15.8	SMBG
Nakamura et al. [72]	Dexcom G4 Platinum	13	GS
Rodbard et al. [71]	MiniMed 640G-E2	14.2	GS
Garg et al. [73]	MiniMed 670G-S3	10.3	GS
Hansen et al. [11]	FreeStyle Libre System	16.7	SMBG
Slover et al. [70]	Guardian-Connect	10.9	SMBG

The article of Gross et al. [74], which studied the CGMS accuracy, provided the MARD during rest periods for Adolfsson et al. [4], 18%. In the case of Moser et al. [33], this value was assigned by Mastrototaro et al. [69], which was 15.8%. Nakamura et al. [72] provided the MARD during rest periods for the Dexcom G4, 13%, used in Bally et al. [34], Aberer et al. [37] and Steineck et al. [50]. Garg et al. [73] provided a value of 10.3% for the MARD during rest periods of the MiniMed 670G-S3, which was compatible with the sensor used in Jayawardene et al. [39]. The value for the FreeStyle Libre System is needed to complement the studies by Aberer et al. [37], Moser et al. [63] and Fokkert et al. [67]. According to Hansen et al. [11], this value is 16.7%. Slover et al. [70] gave the MARD during rest periods for the Guardian-Connect, 10.9%, which can be used to complement the study by Fokkert et al. [67]. Finally, the MiniMed 640G-E2 value, needed for Aberer et al. [37], was provided by Rodbard et al. [71], 14.2%.

**Figure 1:**
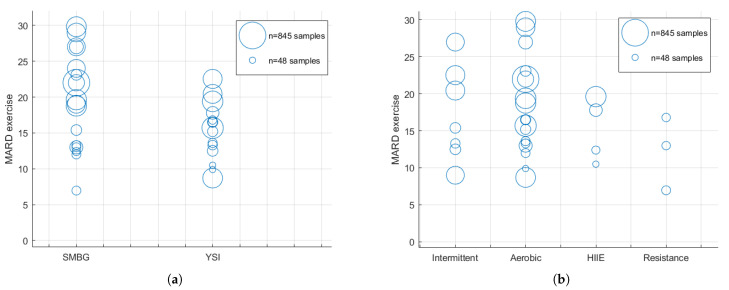
Panel (**a**) shows the MARD for the exercise periods according to the reference used. The SMBG column displays data from studies where self-monitoring blood glucose samples were used to calculate the CGM accuracy. The YSI column stands for those studies that used gold-standard reference methods (YSI or equivalent) to calculate the CGM accuracy. Panel (**b**) displays MARD data for the exercise periods grouped according to the type of exercise. The radius of each bubble is proportional to the number of samples used for the computation of MARD values.

In summary, from the original 54 sources listed in Table 1, sixteen provided MARD data during exercise. These sources are highlighted in Table 1 as shaded rows. These MARD values are displayed in Figure 1 grouped by glucose reference (Figure 1a) and type of exercise (Figure 1b) in order to facilitate visual inspection of the data.

**Figure 2:**
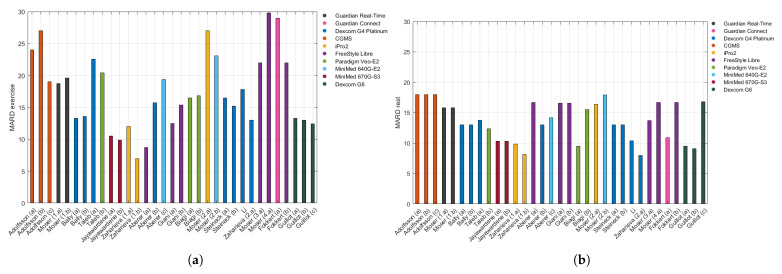
Panel (**a**) shows the MARD for the exercise periods in chronological order of the publication, coloured according to the CGM system used, and Panel (**b**) depicts the MARD of the rest periods, presented in the same way.

Even though the CGM accuracy improved overall with time, it is not trivial to conclude the same about the CGM accuracy during exercise periods. In Figure 2, data are displayed in chronological order in order to better visualize the technological advancements over time.

Figure 2 shows the MARD for exercise periods coloured according to the sensor used in each study:

- CGMS: Medtronic MiniMed Inc., Northridge, CA, USA.
- Guardian Real-Time: Medtronic MiniMed Inc., Northridge, CA, USA.
- Guardian Connect: Medtronic MiniMed Inc., Northridge, CA, USA.
- Dexcom G4 Platinum: Dexcom Inc., San Diego, CA, USA.
- iPro2: Medtronic MiniMed Inc., Northridge, CA, USA.
- FreeStyle Libre System: Abbott Diabetes Care, Maidenhead, UK.
- Paradigm Veo-E2: Medtronic MiniMed Inc., Northridge, CA, USA.
- MiniMed 640G-E2: Medtronic MiniMed Inc., Northridge, CA, USA.
- MiniMed 670G-S3: Medtronic MiniMed Inc., Northridge, CA, USA.
- Dexcom G6: Dexcom Inc., San Diego, CA, USA.

Assuming a relationship exists between CGM accuracy data for the rest periods from Table 2 and those originally listed in Table 1, it was possible to represent MARD data in Figure 2b as an analogous figure to Figure 2a.

**Figure 3:**
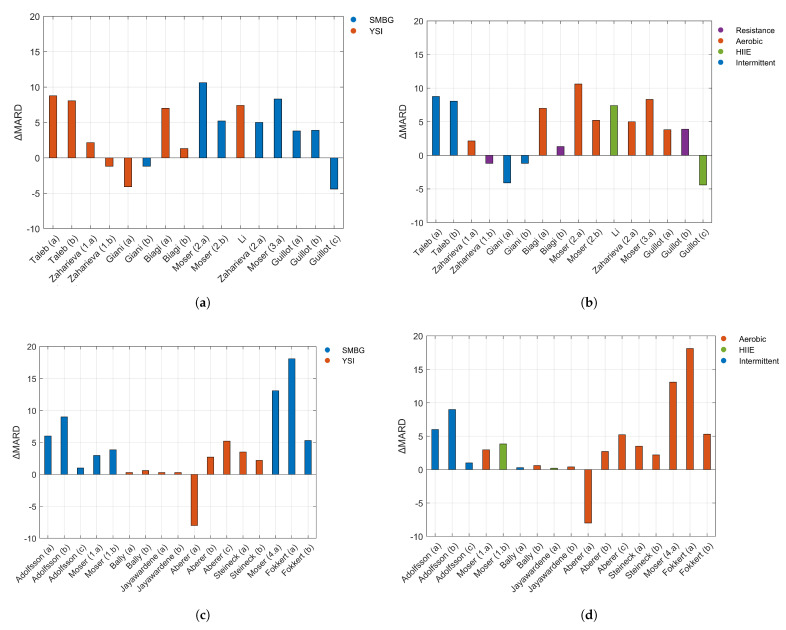
Panels (**a**,**b**) display data from studies that reported the MARD for both exercise and rest. Panel (**a**) shows ΔMARD according to the reference used, and Panel (**b**) shows the same data coloured according to the type of exercise. Panels (**c**,**d**) display data from studies that reported MARD values for exercise periods, but did not provide MARD values for resting periods. In order to calculate ΔMARD for those studies, MARD values for exercise were paired with the MARD from the sources in Table 2. Panel (**c**) displays ΔMARD according to the reference used, and Panel (**d**) presents the same data coloured according to the type of exercise. In Panels (**a**,**c**), YSI stands for those studies that used gold-standard reference methods (YSI or equivalent) to calculate the CGM accuracy.

However, only nine studies originally provided both the MARD during exercise and rest period. For those studies, ΔMARD was calculated to provide a relative measurement of accuracy caused only by the effect of the exercise, as seen in Table 1. In order to keep the flow of information similar to what has already been shown, the data were organized into two different graphs, with the colour palette depending on the reference used (Figure 3a) or the type of exercise (Figure 3b). Positive ΔMARD values indicate a larger error for a particular study during exercise compared to the baseline estimation error of that device and trial.

In order to aggregate the ΔMARD for data from Table 2 and those studies from Table 1 that did not provide MARD for the rest periods, the data are gathered into two new plots: according to the glucose reference used (Figure 3c) and the type of exercise (Figure 3d).

Figure 3a,b is analogous to Figure 3c,d, respectively, in which they are grouped according to the same factor. However, it was preferred not to represent them together in a unique graph, since it is important to remark that the MARD for the rest periods was obtained from different sources and may include a different bias from the MARD during exercise.

### 3.2. Meta-Analysis

The data used in the analysis are the MARD, the number of observations and the Standard Deviation (SD) during the exercise and rest period. In those articles for which the rest data were obtained from external papers (Table 2), the same data were assumed by different control groups. Therefore, in order to avoid “double-counting” the participants in the control group, the pooled effect size of those data was synthesized.

Some articles did not provide the SD, but provided the Interquartile Range (IQR), so in order to make them comparable to the rest, the formula proposed in the Cochrane Handbook [76] and Wan et al. [77] was used: $SD=(Q_{3}-Q_{1})/1.35$, where $Q_{3}$ and $Q_{1}$ stand for the third and first quartiles (75 and 25%). Adolfsson et al. [4] and Fokkert et al. [67] did not provide the SD or IQR of the CGM accuracy; thus, they were excluded from the analysis.

There were two articles that did not provide the SD or IQR of the CGM accuracy, Adolfsson et al. [4] and Fokkert et al. [67]; thus, they had to be excluded from the analysis.

Another method [76] for obtaining the SD is to use the Confidence Interval (CI) values provided: $SD=\sqrt{N}\cdot \left(CImax-CImin\right)/3.92$, *N* being the number of samples. This method was applied for the case of Guillot et al. [68]. In that same article, only the median value for the absolute relative difference of the rest samples was reported. Thus, it was necessary to calculate the mean by applying the approximation proposed at Wan et al. [77].

A subgroup meta-analysis, taking into account the data from Table 1 and Table 2 to populate with enough data each analysed subgroup, was done to factor the influence of the glucose reference in the exercise effect on the MARD, obtaining a *p*-value $p_{\alpha }=0.1023$. Figure 1b shows the MARD value grouped by type of exercise, which could also be a differentiating factor that influenced the MARD during exercise (aerobic/resistance/intermittent/HIIE). Taking that into account, a new subgroup meta-analysis was performed, but now factoring the type of exercise. A *p*-value $p_{\beta }=0.5729$ was calculated.

**Figure 4:**
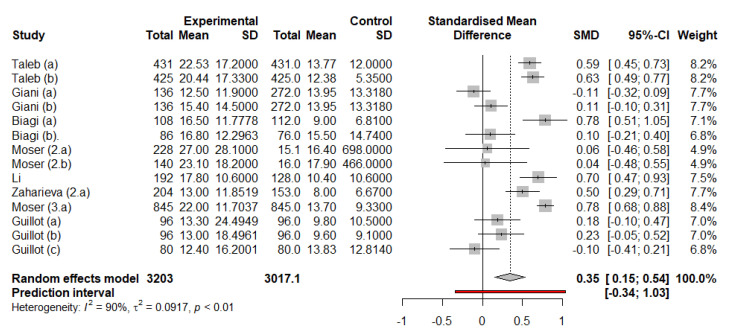
Meta-analysis using the random effects model, including the corresponding forest plot. The above-mentioned outliers were excluded. SMD stands for Standardised Mean Difference.

Considering simultaneously the accuracy data from both exercise and rest periods as extracted in Table 1, without taking into account data from Table 2, a relative metric for sensor accuracy was calculated in ΔMARD, obtaining $p_{\gamma }=0.8244$ for the estimation of its mean value. Data from Zaharieva et al. [40] were found to be quantitatively different than the rest of the data analysed; the reported standard deviation for that study was an order of magnitude lower than that of any other analysed papers, probably due to the retrospective calibration method of estimation of the iPro2 device. This in turn could affect the standardised mean difference (main metric for the meta-analysis), making it the highest and lowest values of all those in the analysis, greatly influencing in return its outcome. Those numbers were extreme and were considered outliers, and therefore excluded from the analysis. The resulting analysis after removing those data is shown in Figure 4, with a *p*-value $p_{\delta }=0.0018$.

## 4. Discussion

Selecting the variables for the grouping and visualization of the MARD data was challenging. The fact that many studies have a similar length and intensity produced an overlap of most studies in these factors, rendering those studies impossible to separate.

The CGM device is one of the more relevant factors considered, as it changes among the different studies and years of literature. As technology evolves, the MARD is expected to be reduced with newer generations of CGM being introduced to the market. Thus, in general terms, the more recent sensor used, the better the technology and more accurate the CGM devices are. As may be observed in Figure 2a (the MARD exercise according to the sensor used), that trend can be appreciated: the oldest Medtronic sensors, CGMS (2000–2002) and Guardian Real-Time (2005), presented more error than the one integrated into the Minimed 670G-S3 (2016). The FreeStyle Libre, a Flash Glucose Monitoring (FGM) device requiring manual scanning, usually provided less accurate measurements [55,78].

There seems to be a mismatch between the marketed date of the CGM devices and their use in the studies screened. For example, Li et al. [3] (2019) and Zaharieva et al. [54] (2019) used the Dexcom G4 Platinum (2014). Even further, some studies used devices of more than 10 years old. For example, the Guardian-Real Time was used in Iscoe et al. [7] from 2006 and in the study of 2017 of Gawreick et al. [38]. It is likely that this date mismatch affected the analysis of the CGM accuracy over time. Perhaps the use of an obsolete sensor would not be the reason for less accurate results, but its influence cannot be ruled out either.

It is remarkable to find a lack of new-generation sensors used in exercise studies, such as Dexcom G5 or G6, even in more recent studies, when they present improved accuracy compared to previous generations according to the manufacturers. Zaharieva et al. [54] and Guillot et al. [68] are the only studies that provided MARD values using Dexcom G5 (MARD exercise 13%) and Dexcom G6 (MARD exercise 13.3%, 13% and 12.4%), respectively.

Regarding MiniMed 640G-E2, it was not possible to draw any conclusions, as it was used in only two papers. Other devices, like Medtronic 670G-S3, Dexcom G4 Platinum and Paradigm Veo-E2, presented lower errors. No clear relationship could be established between the CGM accuracy of these devices and the analysed factors.

The iPro2 initially seemed to be the most accurate sensor for exercise periods. It seems to be better than every other CGM system under study. The limitation with iPro2 CGM, however, is that glucose values are not reported in real-time, since they use a retrospective calibration algorithm, and as such, it can only be used for retrospective analysis such as the one presented in [40].

At first glance when analysing Figure 1a, which shows the MARD under exercise classified per reference glucose used, studies using SMBG seem to have a greater MARD than those using YSI. To clarify this hypothesis, a meta-analysis was done on the data available. The *p*-value $p_{\alpha }$, which was obtained from the subgroup meta-analysis between the studies that used SMBG and YSI, shows that the type of reference could not be determined as a relevant factor to the difference in the MARD between groups, as it was higher than 0.05. The type of exercise factor also resulted in being non-conclusive, as a *p*-value $p_{\beta }$ of 0.5729, also greater than 0.05, was obtained from its subgroup meta-analysis.

The fact that a non-significant *p*-value $p_{\gamma }$ was obtained for the initial hypothesis that the exercise is detrimental to the MARD does not allow the above-mentioned hypothesis to be corroborated and is contrary to what was previously observed. This led to a new meta-analysis displayed in the forest plot in Figure 4, which resulted in a statistically significant *p*-value $p_{\delta }=0.0018$, confirming that CGM during exercise periods is less accurate. This has important implications for diabetes management, since people with diabetes must be aware about the possible inaccuracy of glucose sensing devices during exercise (i.e., periods of increased glucose fluctuations).

On the other hand, the analysis of the MARD in Figure 3a,b ΔMARD according to the reference use and type of exercise) provided interesting conclusions. It can be appreciated that the accuracy was lower during intermittent and aerobic exercises. Only two studied HIIE, thus no tendency can be determined. One of the cases presented a negative ΔMARD value, which means that the MARD for exercise was lower (12.4%) than the pre-exercise value (16.8%). With respect to the resistance exercises, a greater accuracy of the sensors can be observed, as it was concluded in individual studies that compared resistance exercise and other exercises [8,46]. One of the cases also presented a negative ΔMARD value, the MARD for exercise being 6.96% and the MARD for rest being 8.15%. This case did not alter the trend observed in the other studies.

Comparing the graphs of ΔMARD according to the reference used, in Figure 3a,c (left top and bottom graphs), it is worth noting that negative values of ΔMARD occur mostly when the reference is SMBG. It is also apparent that positive values for SMBG in both figures range between 2% and 9%. Regarding the graphs of ΔMARD according to the type of exercise, in Figure 3b,d, negative values do not coincide with the same type of exercise: in the upper graph, they correspond to intermittent and resistance activity, and in the bottom graphs to aerobic. In both figures, the positive values for intermittent exercise lay between 6% and 9%. Aerobic exercise in Figure 3d seems to show higher values, reaching a maximum of 18.1%, while in Figure 3b, the highest value is 8.3%.

For individuals with type 1 diabetes, aerobic exercise typically leads to the greatest risk of hypoglycaemia. CGM accuracy during exercise periods is therefore critical for detecting and potentially treating these situations. Patients need to know that they can rely on CGM systems during any kind of situation with a fast and accurate response. Further work needs to be done exploring the impact of CGM accuracy on aerobic exercise, and possibly correcting that impact, as proposed by Laguna et al. [8].

It is particularly interesting to consider the implications of the CGM accuracy on closed-loop artificial pancreas systems, which are the technological vanguard in T1D treatments. In Huyett et al. [79], a comparison of glucose sensing dynamics vs. closed loop performance was performed, showing that a lower sensor lag (and in turn, a lower MARD) yields better treatment by a closed-loop algorithm after meal ingestion. It is thus paramount for the adequate treatment of people with diabetes to ensure the accuracy of CGM during every stage of their lives, particularly during periods of great glycemic variability. This is especially critical during periods of high hypoglycaemic risk such as exercise bouts, as stated above.

In the literature analysed in this review, it was hard to establish a clear relationship between MARD and any factors relevant to either the exercise or the underlying diabetes condition. Despite the amount of studies that complied with the selection conditions, only some of them provided the MARD during exercise, and even fewer reported the MARD during rest periods. In other cases, the MARD was not provided as a mean value, but instead, the median ARD [2,26,53] or Mean Absolute Difference (MAD) [23,80] was reported. The main reason could be that the studies did not focus on the accuracy of the CGM during the exercise. Some of them were focused on the analysis of the heart rate in the different exercises and post-exercise periods [31,59,81], time spent in hypoglycaemia [42,60,65] or glucose levels [41,51,66].

Therefore, this work encourages further detail of the CGM accuracy in exercise periods in future studies, facilitating the proposal of a solution to solve CGM errors during periods with high risk of hypoglycaemia. Given that the blood glucose reference was not found to relevantly influence the effect of exercise on the accuracy of the sensors evaluated, we acknowledge the clear benefits of favouring SMBG devices (which keep improving in accuracy [82]) over gold-standard reference measurements, which are limited to laboratory analysis and IV blood sampling. It must be remarked that CGM devices are calibrated using SMBG, and accuracy cannot be expected to be better than that of the calibration device used. Furthermore, SMBG allows for more flexible outpatient exercise monitoring protocols, which tend to provide richer sets of data over more diverse cohorts of patients. Additionally, SMBG also facilitates gathering accuracy data from resting ambulatory periods at lower costs than those in a clinic environment. In addition, many of the in-clinic studies analysed are often preceded by days of free living conditions of the patients after CGM insertion. CGM data from these days, coupled with several daily SMBG measurements, could constitute the baseline MARD that is missing in many of the works that report the MARD for exercise, without increasing the cost of the experiments.

However, for those studies in which the primary goal is discerning the influence of exercise on the CGM accuracy, more detailed guidelines should be discussed. The studies analysed in this work reported widely different sample sizes with MARD values during rest periods and those with exercise. These differences in sample sizes could hinder the comparison of MARD values and the computation of the exercise effect. As recommended by Danne et al. [83], head-to-head studies are encouraged to avoid sample mismatches in the MARD estimations. As for the number of samples to collect, Reiterer et al. [84] stated that confidence in the estimation of the CGM accuracy greatly depends on the sample size of the study. Thus, ideally, the sample size must be maximized. In practice, it is often unreasonable to ask more for than 60 min of exercise in a trial from a patient (although it depends on the cohort). Additionally, if the MARD were to be monitored using SMBG, the patient would be asked to use a finger strip every several minutes, which is also unrealistic for long periods of exercise. Therefore, a compromise must be achieved: we propose that, for MARD characterization purposes, more than 45 min of exercise for each visit are scheduled, and at least four reference glucose samples are collected. The trial number of samples can be maximized by repeating the exercise visits with the same or different patients.

In summary, the main findings of this work are threefold: (1) exercise negatively affects CGM accuracy; (2) no clear statistical influence on the CGM accuracy was found for factors such as type of exercise or glucose reference used; and (3) few studies simultaneously reported CGM accuracy for both exercise and rest periods, which makes the analysis of the influence of exercise on CGM errors difficult.

## 5. Conclusions

In this review, an analysis of published articles that study people with T1D using CGM during exercise was performed. The results of the meta-analysis indicate that the accuracy of the monitoring devices is negatively affected by the exercise periods. This is of particular interest to know for both researchers, who could take it into consideration when designing new exercise experiments, and patients or clinicians who try to manage diabetes, who must consider the presence of larger CGM errors during periods of exercise.

MARD data from multiple studies were pooled, visualized and statistically compared. It was shown that no clear statistical difference can be found in the precision of sensors for factors such as the blood glucose measurement method or the type of exercise. More modern sensors are expected to be more accurate during periods of exercise; however, it was found that very few studies for exercise have been done using the latest generation of sensors, to our surprise.

## Figures and Tables

**Table 1:** Reported data in chronological order of publication of the surveyed studies that carried out exercise trials. GS stands for Gold Standard glucose reference. ΔMARD $=MARD_{e}-MARD_{r}$ where $MARD_{e}$ is the MARD of the samples within exercise periods and $MARD_{r}$ is the counterpart for the samples during rest periods. Asterisks (*) indicate that $MARD_{r}$ was extracted from the articles mentioned in Table 2. The shaded rows correspond to those papers that reported necessary data to assess sensor accuracy during exercise, 17 in total. The labels that will later be used in the graphics to uniquely identify a study and branch have also been added to the MARD exercise column.

Source	Year	Patients	Exercise Samples	Exercise	Sensor Used	MARD Rest (%)	MARD Exercise (%)	ΔMARD	Reference
Iscoe et al. [7]	2006	5	–	Cycling	Guardian Real-Time	–	–	–	SMBG
Fayolle et al. [22]	2006	9	–	Cycling	GlucoDay	–	–	–	GS
Adolfsson et al. [23]	2008	12	–	Scuba diving	CGMS	–	–	–	GS
Riddell et al. [24]	2011	25	–	Sport Camps	Guardian Real-Time	–	–	–	SMBG
Adolfsson et al. [4]	2011	18	1135	Soccer	CGMS	*18	24 (a)	6	SMBG
		20		Skiing			27 (b)	9	
		21		Golf			19 (c)	1	
Herrington et al. [5]	2012	12	–	Cycling	Dexcom Seven Plus	–	–	–	GS
Yardley et al. [25]	2012	12	–	Aerobic and resistance	Medtronic Gold CGM	–	–	–	GS
Kumareswaran et al. [2]	2012	12	–	Walking	Freestyle Navigator	–	–	–	GS
Kumareswaran et al. [26]	2013	10	–	Walking	Freestyle Navigator	–	–	–	GS
Yardley et al. [27]	2013	12	–	Aerobic and resistance	Medtronic Gold CGM	–	–	–	GS
Radermecker et al. [28]	2013	10	–	Cycling	Guardian Real-Time	–	–	–	GS
Yousef et al. [29]	2014	12	–	Skydiving simulation	iPro2	–	–	–	SMBG
Campbell et al. [30]	2015	9	–	Running and simulate game-play activities	Medtronic Gold CGM	–	–	–	GS
Moser et al. [31]	2015	8	–	Aerobic	Guardian Real-Time	–	–	–	SMBG
van Dijk et al. [32]	2016	10	–	Walking	iPro2	–	–	–	SMBG
Moser et al. [33]	2016	7	489	Continuous cyclometer HIIE cyclometer	Guardian Real-Time	*15.8	18.76 (1.a) 19.63 (1.b)	2.96 3.83	SMBG
Bally et al. [34]	2016	10	108 100	Intermittent cycling Continuous cycling	Dexcom G4	*13	13.3 (a) 13.6 (b)	0.3 0.6	GS
Taleb et al. [35]	2016	17	431 425	Intermittent	Dexcom G4 Platinum Paradigm Veo (Enlite2)	13.77 12.38	22.53 (a) 20.44 (b)	8.76 8.06	GS
Moser et al. [33]	2016	7	–	Cycling	Guardian Real-Time	–	–	–	GS
McAuley et al. [36]	2016	14	–	Cycling	Paradigm Veo (Enlite2)	–	–	–	GS
Aberer et al. [37]	2017	12	462 540 502	Cycling	FreeStyle Libre Dexcom G4 Platinum MiniMed 640G (Enlite2)	*16.7 *13 *14.2	8.7 (a) 15.7 (b) 19.4 (c)	−8 2.7 5.2	GS
Gawrecki et al. [38]	2017	29	–	Walking	Guardian Real-Time	–	–	–	SMBG
Jayawardene et al. [39]	2017	12	48 48	HIIE cycling Cycling	MiniMed 670G (Sensor3)	*10.3	10.5 (a) 9.9 (b)	0.2 0.4	GS
Zaharieva et al. [40]	2017	12	96 96	Aerobic Resistance	iPro2	9.86 8.15	12 (1.a) 6.96 (1.b)	2.14 −1.19	SMBG
Reddy et al. [41]	2017	10	–	Aerobic and resistance	Dexcom G4 Platinum or G5	–	–	–	SMBG
Quirós et al. [42]	2018	5	–	Aerobic and resistance	Paradigm Veo (Enlite2)	–	–	–	GS
Larose et al. [43]	2018	22	–	Cycling	Dexcom G4 Platinum	–	–	–	SMBG
Giani et al. [6]	2018	17	136 136	Intermittent	FreeStyle Libre	16.6	12.5 (a) 15.4 (b)	−4.1 −1.2	GS SMBG
Aronson et al. [44]	2018	17	–	HIIE	Dexcom G4 Platinum	–	–	–	GS
Reddy et al. [45]	2018	10	–	Aerobic	Dexcom G4 Platinum	–	–	–	SMBG
Biagi et al. [46]	2018	6	108 86	Aerobic Resistance	Paradigm Veo (Enlite2)	9.5 15.5	16.5 (a) 16.8 (b)	7 1.3	GS
Abdulrahman et al. [47]	2018	4	–	Rugby training	Paradigm Veo (Enlite2)	–	–	–	SMBG
Castle et al. [48]	2018	20	–	Aerobic	Dexcom G5	–	–	–	SMBG
Moser et al. [49]	2018	10	228 140	Aerobic	iPro2 MiniMed 640G (Enlite2)	16.4 17.9	27 (2.a) 23.1(2.b)	10.6 5.2	SMBG
Steineck et al. [50]	2019	13	2660	Cycling	Dexcom G4 Platinum	*13	16.5 (a) 15.2 (b)	3.5 2.2	GS
Burckhardt et al. [51]	2019	14	–	Aerobic	Dexcom G5	–	–	–	SMBG
Forlenza et al. [52]	2019	12	–	Aerobic	Dexcom G4 (505)	–	–	–	SMBG
Larose et al. [53]	2019	22	–	Aerobic	Dexcom G4 Platinum	–	–	–	SMBG
Li et al. [3]	2019	17	192	HIIE	Dexcom G4 Platinum	10.4	17.8	7.4	GS
Zaharieva et al. [54]	2019	17	204	Aerobic	Dexcom G4(505) or G5	8	13 (2.a)	5	SMBG
Moser et al. [55]	2019	10	845	Cycling	Freestyle Libre	13.7	22 (3.a)	8.3	SMBG
Zaharieva et al. [56]	2019	12	–	Resistance	iPro2	–	–	–	SMBG
Eshghi et al. [57]	2019	12	–	Resistance	iPro2	–	–	–	SMBG
Steineck et al. [58]	2019	14	–	Cycling	Dexcom G4 Platinum	–	–	–	SMBG
Gawrecki et al. [59]	2019	16	–	Football	Guardian Connect	–	–	–	SMBG
Lee et al. [60]	2019	12	–	Cycling	FreeStyle Libre Pro	–	–	–	SMBG
Moser et al. [61]	2019	10	–	Cycling	FreeStyle Libre	–	–	–	SMBG
Scott et al. [62]	2019	14	–	Cycling	Dexcom G4 Platinum	–	–	–	SMBG
Moser et al. [63]	2019	14	470	Cycling	FreeStyle Libre	*16.7	29.8 (4.a)	13.1	SMBG
Scott et al. [64]	2019	14	–	HIIE and MICT	Dexcom G4 Platinum	–	–	–	SMBG
McCarthy et al. [65]	2020	16	–	Cycling	Dexcom G6	–	–	–	Unknown
Brockman et al. [66]	2020	23	–	Resistance	Medtronic Gold CGM iPro2	– –	– –	–	GS
Fokkert et al. [67]	2020	14	414 311	Mountain biking	Guardian Connect FreeStyle Libre	*10.9 *16.7	29 (a) 22 (b)	18.1 5.3	SMBG
Guillot et al. [68]	2020	24	96 96 80	Aerobic Resistance HIIE	Dexcom G6	9.5 9.1 16.8	13.3(a) 13 (b) 12.4 (c)	3.8 3.9 −4.4	SMBG

## Abbreviations

The following abbreviations are used in this manuscript:

T1D	Type 1 Diabetes
CGM	Continuous Glucose Monitoring
IDF	International Diabetes Federation
FGM	Flash Glucose Monitoring
MARD	Mean Absolute Relative Difference
MAD	Mean Absolute Difference
HIIE	High Intensity Interval Exercise
MICT	Moderate-Intensity Continuous Training
SMBG	Self-Monitoring Blood Glucose
NCBI	National Center for Biotechnology Information
NLM	National Library of Medicine
CG	Capillary Glucose
GS	Gold Standard
